# Experiences of GP trainees in undertaking telephone consultations: a mixed-methods study

**DOI:** 10.3399/bjgpopen20X101008

**Published:** 2020-02-05

**Authors:** Umar Chaudhry, Judith Ibison, Tess Harris, Imran Rafi, Miles Johnston, Tim Fawns

**Affiliations:** 1 Clinical Teaching Fellow, Population Health Research Institute (PHRI), St George's University of London (SGUL), London, UK; 2 Reader in Primary Care, Institute of Medical and Biomedical Education (IMBE), St George's University of London (SGUL), London, UK; 3 Professor of Primary Care Research, Population Health Research Institute (PHRI), St George's University of London (SGUL), London, UK; 4 Senior Lecturer in Primary Care Education, Institute of Medical and Biomedical Education (IMBE), St George's University of London (SGUL), London, UK; 5 General Practitioner, Parliament Hill Medical Centre, London, UK; 6 Lecturer in Clinical Education, Medical Education Department, Edinburgh Medical School, The University of Edinburgh, Edinburgh, UK

**Keywords:** general practice, remote consultation, telephone consultations, education, quantitative evaluation, qualitative research

## Abstract

**Background:**

Primary care telephone consultations are increasingly used for patient triage, reviews, and providing clinical information. They are also a key postgraduate training component yet little is known about GP trainees’ preparation for, or experiences and perceptions of, them.

**Aim:**

To understand the experiences, perceptions, and training of GP trainees in conducting telephone consultations.

**Design & setting:**

A mixed-methods study was undertaken of North Central and East London (NCEL) GP trainees.

**Method:**

A cross-sectional electronic survey of trainees was performed with subsequent semi-structured interviews. Survey data were analysed using descriptive statistics, and qualitative data using thematic analysis.

**Results:**

The survey response was 16% (*n* = 100/618), and 10 participated in semi-structured interviews. Trainees felt least confident with complicated telephone consulting, and there was a strong positive correlation between the percentage reporting having received training and their confidence (*R* ^2^ = 0.71, *P*<0.0001). Positive experiences included managing workload and convenience. Negative experiences included complex encounters, communication barriers, and absence of examination. Trainees reported that training for telephone consultations needed strengthening, and that recently introduced audio-clinical observation tools (COTs) were useful. Positive correlations were found between the length of out-of-hours (OOH) but not in-hours training and the level of supervision or feedback received for telephone consultations.

**Conclusion:**

This project sheds light on GP trainees’ current experiences of telephone consultations and the need to enhance future training. The findings will inform a wider debate among stakeholders and postgraduate learners regarding training for telephone consultations, and potentially for other remote technologies.

## How this fits in

Telephone consultations are a vital component in primary care for daily triage, patient reviews, and providing clinical information, but little is known about GP trainees’ experiences and perceptions of telephone consulting. This project sheds light on the experience of GP trainees in conducting telephone consultations, their current learning, feedback, and assessment practices, which need to be strengthened. The findings will inform postgraduate training for both telephone consultations and the use of other remote technologies in primary care.

## Introduction

Telehealthcare, or *'* *the provision of personalised health* *care over a distance* *'*, involves patients transferring data electronically to a clinician, who uses it to develop a personalised management plan.^1^ Telephone consultations are an early example of real-time telehealthcare and can improve patient access and convenience without compromising patient satisfaction.^2,3^ Significantly increasing over the last decade, they now account for up to a quarter of all UK primary care consultations and cover many clinical activities.^1,2,4^ Telephone consultations and technology will play increasingly integral roles in transforming health care, allowing faster triage and better access to help meet population demands.^5^ Recent primary care policy initiatives place significant emphasis on digital technology as a means to improve access to and convenience of advice and care, and this includes through telephone consultations.^6,7^

A recent Cochrane review of interventions to improve clinicians’ telephone consultation skills concluded that telephone communication with patients was generally of low quality, with undergraduate and postgraduate curricula lacking specific training.^8^ While telephone consulting is included within the Royal College of General Practitioners (RCGP) curriculum under several core competencies, including 'communication and consultation' and 'community orientation' domains,^9^ there are concerns that, with telephone consultations, GPs may feel under-skilled and lack the ability to react to non-verbal cues, perform examinations, and manage ethical and/or medico-legal challenges.^10^ Telephone consultations may adversely affect patient safety, owing to less data gathering and rapport building than face-to-face consultations, and they may not reduce workload.^3,11^

There is, therefore, a growing call for further professional development in delivering telephone consultations in order to improve quality and to guide changes in undergraduate and postgraduate training.^12^ Greater understanding of the views and experiences of GPs is needed in training those responsible for shaping and delivering future primary care, particularly in terms of GPs' training needs, curricular requirements, and confidence in handling telephone consultations. Therefore, the main research question of this study is: what are the current experiences of GP trainees in conducting telephone consultations with patients?

## Method

### Study design

This mixed-methods study comprised an initial cross-sectional electronic survey of NCEL GP trainees, followed by qualitative semi-structured telephone interviews, allowing for deeper exploration of themes.^13,14^ An electronic questionnaire using an online survey tool (LimeSurvey) was sent to all 618 GP trainees in NCEL, between February and April 2018. Responders who agreed to participate in a semi-structured interview were contacted between May and October 2018.

#### Questionnaire and semi-structured interview development

The purpose of the questionnaire and semi-structured interviews was to gain an understanding of GP trainees’ perceptions and experiences of telephone consultations, taking account of the range and contexts of their individual training environments. The questions were developed through reviewing the gaps identified in the literature, gaps in knowledge, and the project’s research objectives. A list of essential skills for telephone consultations were considered, based on the current available evidence,^12,15^ and further questions relating to confidence, positive or negative experiences, and in-hours versus OOH practice were developed. There were also questions on demographic details and training information, and space for individual comments. The questionnaire was divided into seven sections and comprised 30 short questions. A 5-point Likert scale was used to measure the degree of confidence or agreement to statements. A focus group composed of five qualified GPs (two of whom were qualified GP trainers) and three GP trainees helped in designing, refining, and finalising both the questionnaire and the semi-structured schedule. Questionnaire findings were later fed into the further development of the interview schedule, allowing the semi-structured interviews to probe deeper and make further sense of questionnaire responses. The questionnaire and semi-structured interview schedule are provided in Supplementary Appendices A and B.

#### Study setting and participants

The initial proposal was to survey a random sample of all GP trainees in London. Only data collection from NCEL was approved, and the resulting sample population at survey commencement (February 2018) was all GP trainees in NCEL London, at any stage of their specialist GP training at one of the 11 NCEL vocational training schemes (VTS). There were no exclusion criteria. The study context incorporated London-based training, including urban and less urban areas. GP trainee consent was implied by return of the electronic questionnaire. Using convenience sampling, trainees who completed the questionnaire and agreed to be contacted for a semi-structured interview were invited to complete a written consent form and participate in an interview.

#### Questionnaire data collection and analysis

GP trainees were invited to complete the questionnaire through an initial email approach, four email reminders, and also by announcements from the programme directors at weekly training.

Levels of confidence for questions were scored of 1–5 (1 signifying ‘strong disagreement’, 3 signifying ‘neither agreement nor disagreement’, and 5 signifying ‘strong agreement’). The software GraphPad Prism (version 6) was used for statistical analyses. Comparisons were made between mean scores at different stages of training using the Mann-Whitney U-test. Linear regression was used to determine correlations between confidence of trainees to work independently and prior training received across different aspects of telephone consultations. Correlations were also sought using Spearman’s rank sum correlations between time spent in-hours and OOH with: (i) overall confidence in telephone consultations; (ii) satisfaction with supervision received; and (iii) satisfaction with feedback received.

#### Semi-structured interview data collection and thematic analysis

Semi-structured interviews were conducted by either telephone or face-to-face, and audio-recorded. They commenced with an introduction and brief study outline. Interviews were roughly 30 minutes each and recordings were transcribed and anonymised. Analysis was undertaken using thematic analysis, following Braun and Clarke’s method, which involved identifying, examining, and coding patterns of responses into categories.^16–18^ The software NVivo (version 12) was used to aid transcript coding and formulating categories. Data collection and analysis were performed concurrently, allowing questions to be modified and themes tested in subsequent interviews. The themes were reviewed for coherence and relevance.

Results from the questionnaire survey and semi-structured interviews are presented in parallel, and analyses of both mixed-method components were synthesised to form conclusions in relation to the overall aim.

## Results

The questionnaire response rate was 16% (*n* = 100/618). Seventy-five per cent (*n* = 75/100) of the responders were female, and a range of age groups, practice sizes, and stages of training was covered from both North Central (NC) and North East (NE) groups (Table 1). Seventeen responders provided contact details to participate in the semi-structured interviews, of whom 10 ultimately agreed to be interviewed; they were at different stages of training and from both NC and NE regions (Table 1). Eight interviews were initially conducted and analysed until theoretical saturation was reached, and a further two interviews allowed for validation of the initial themes generated.

**Table 1.** Demographic findings for survey and semi-structured interviews

Characteristic	Frequency, *n* (%)
**Survey responders (*n* = 100)**		
**Sex**	Male	25 (25)
	Female	75 (75)
**Age, years**	25–29	50 (50)
	30–34	38 (38)
	≥35	12 (12)
**Practice population**	0–5000	9 (9)
	5001–10000	37 (37)
	10 001–15 000	34 (34)
	15 001–20 000	18 (18)
	>20 000	2 (2)
**Stage of training**	ST1	32 (32)
	ST2	29 (29)
	ST3	36 (36)
	ST4	3 (3)
**Current VTS region**	North Central	62 (62)
	North East	62 (62)
**Semi-structured interview participants (*n* = 10)**		
**Stage of training**	ST1	3 (30)
	ST2	1 (10)
	ST3	4 (40)
	ST4	2 (20)
**Current VTS region**	North Central	7 (70)
	North East	3 (30)

ST = specialty training (stage). VTS = vocational training scheme

### Confidence in undertaking particular tasks in telephone consultations

**Figure 1.**
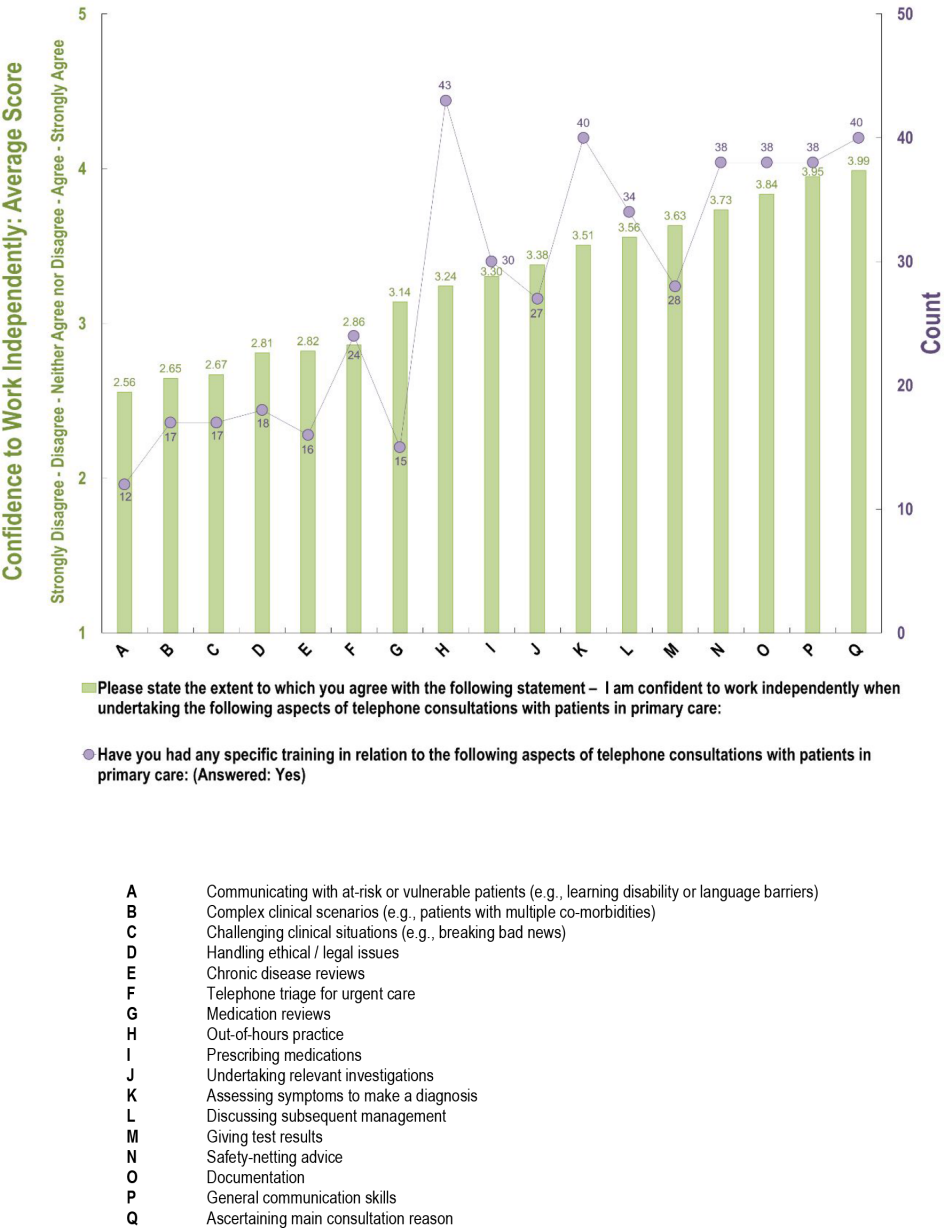
Confidence to work independently and prior training received across different aspects of telephone consultations

The distribution of trainee confidence to work independently, and mean confidence levels of responders in 17 aspects of telephone consultations are tabulated in ascending order (Figure 1). GP trainees rated least confidence with the following aspects of telephone consultations: communicating with at-risk or vulnerable patients (for example, those with learning disability or language barriers); complex clinical scenarios (for example, patients with multiple comorbidities); and challenging clinical situations (for example, breaking bad news). On the other hand, general communication skills and documentation were seen as aspects of telephone consultations that GP trainees felt most confident to undertake independently.

**Figure 2.**
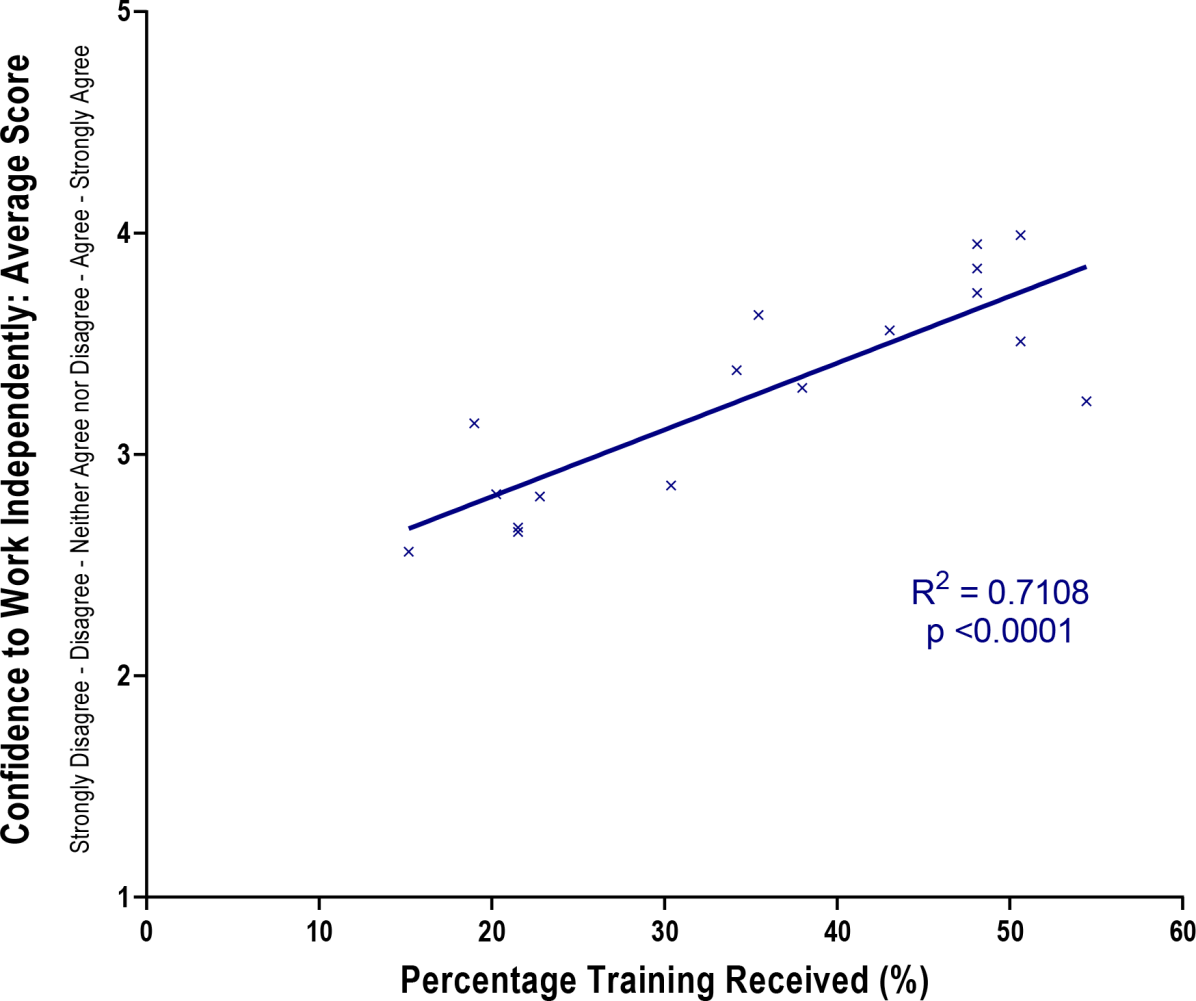
Relationship between percentage training received and the confidence to work independently for telephone consultations.

GP trainees were also asked about specific training in relation to these 17 aspects and this was charted alongside their level of confidence as counts to those who responded ‘yes’ (Figure 1). Generally, those aspects that GP trainees had the least amount of training in, were also those where they felt least confident in working independently, and vice versa. Figure 2 demonstrates that there was a strong positive correlation (*R* ^2^ = 0.71, *P*<0.0001) between whether or not trainees had received training and their confidence to work independently.

One emergent theme from the semi-structured interviews was the positive impact of experience consulting by phone on confidence. For example:

*'I think actually if you do telephone consulting, in practice, certainly, it's just the more you do it the more you get used to it.'* (Interview 1, ST4)

*'* [...] *the more you practise that skill, the more you I suppose reflect on when it goes wrong, then you become more confident and better at it in both ways.'* (Interview 3, ST3)

### Positive and negative experiences

**Figure 3.**
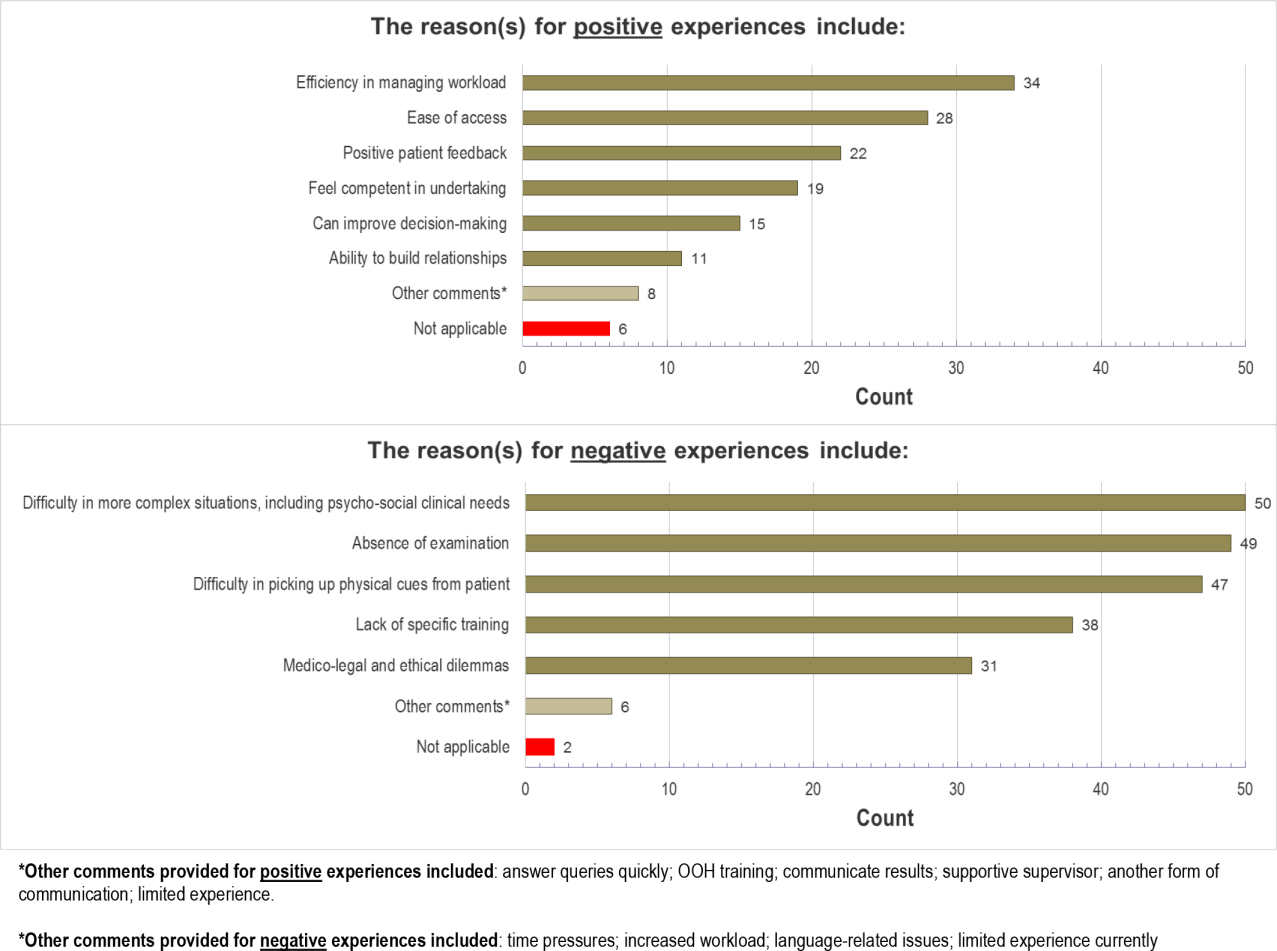
Reasons for positive and negative experiences with telephone consultations from questionnaire data.

The questionnaire data shown in Figure 3, supported by the qualitative data, describe the main reasons selected by GP trainees for positive experiences of telephone consultations: their own efficiency in managing workload; ease of access for patients; and positive patient feedback. During the interviews, these advantageous patient-related and doctor-related factors of convenience, reduced workload, and more efficient decision-making were highlighted:

*'* [...] *definitely reduces your workload* […] *much more efficient because you take that history on the phone.'* (Interview 2, ST3)

*'* [...] *with emergency problems that need to go in to hospital and you know it saves them the time of coming in.'* (Interview 3, ST3)

*'* [...] *some of the stuff can be dealt with remotely* […] *they take like two minutes to do.'* (Interview 5, ST3)

There was an association between trainees’ seniority and their agreement with a statement about an overall positive experience in undertaking telephone consultations with primary care patients. Trainees in years 3–4 (specialty training [ST] 3–4s) were more likely to agree with this statement (mean score 3.8) compared with trainees in years 1–2 (ST1–2s) (mean score 2.9, *P*<0.0001).

Similarly, the main reasons for negative experiences given in the interviews, were the difficulty in more complex consultations (for example, risk assessment), absence of non-verbal cues, and absence of examination with the consequent difficulty in picking up physical cues from patients (Figure 3):

​*'* [...] *it's really hard to safety net* […] *you end up having to bring someone in.'* (Interview 1, ST4)

*'* [...] *if they are hard of hearing, or English isn't their first language* […] *or the line's fuzzy.'* (Interview 4, ST3)

*'* […] *skewed picture* […] *no body language, no visible clues.'* (Interview 4, ST3)

*'* [...] *higher degree of uncertainty* […] *lack of kind of physical feedback, lack of examination, it's more risky* *.'* (Interview 7, ST4)

Difficulties in terms of privacy, confidentiality and particularly differences between the patient and doctor’s agenda were also highlighted as important themes in some of the interviews, which were not raised in the earlier questionnaires.

*'* [...] *their sort of agenda* […] *that can be quite difficult when you're* […] *trying to manage expectations of what can and can't be done through the telephone consult.'* (Interview 8, ST1)

*​'* [...] *trying to understand the patient's agenda is a skill* […] *even more difficult to achieve, on a telephone conversation* […] *skills to understand what's appropriate and what's inappropriate.'* (Interview 9, ST1)

### Experiences of training

All trainees, regardless of stage of training and region, agreed or strongly agreed with statements relating to gaps in their learning and a need for training for undertaking telephone consultations to be strengthened. However, a key positive aspect of training was the recent introduction of audio-COTs for the learner portfolios, and trainees as part of the qualitative interviews emphasised their benefits:

*​* *'* [...] *well I think audio* *-* *COT* *s have been brilliant* […] *I've used them three or four times now* […] *I think that's been a real plus* *.'* (Interview 7, ST4)

*'* [...] *if a COT could also just be someone listening to you do a few telephone consultations, in primary care, I think that would be good.'* (Interview 1, ST4)

Based on the questionnaire responses for current training and supervisor feedback in relation to telephone consultations, the most common form of training that GP trainees indicated that they had received was trainer-led teaching with feedback provided by the supervisor. However, a similar number claimed to receive no training and feedback, and this fits with the qualitative narrative of requesting more training and feedback:

*'I think definitely some training earlier on* […] *more specific, explicit training needs would be better for us to train up our skills.'* (Interview 6, ST1)

*'I guess getting feedback* [...] *I haven't had the opportunity to really have people listen to me and then suggest what they would do differently, which I think would be helpful.'* (Interview 10, ST2)

#### In-hours versus out-of-hours practice

A further finding from across the questionnaires and interviews was the perceived differences between the adequacy of training for telephone consultations in the different service context and case-mix of in-hours and OOH clinical services. Given the differing experiences in-hours and OOH, the authors evaluated whether the length of experiences in each had an effect on the overall impact in terms of confidence, supervision, and feedback received. There were weakly positive correlations (*P*<0.01) between experience both in terms of length of time or number of contacts and building overall confidence in telephone consulting both in-hours and OOH (Table 2). There were also weakly positive correlations (*P*<0.01) between greater OOH experience (in terms of length of training and average number of contacts) and greater satisfaction in terms of both supervision and feedback received, which was not seen for in-hours training (Table 2). The semi-structured interviews provided some explanations for this, with one participant reporting their OOH training as follows:

*'* [...] *had a lot of supervised sessions* […] *so we've been observed undertaking phone calls … and been given feedback*.' (Interview 2, ST3)

Other trainees described in-hours training as follows:

*'* [...] *in-house telephone consults, can be a bit harder* […] *friction between the patient's agenda and what can and can't be done.'* (Interview 8, ST1)

*‘* [...] *a set number of hours* […] *but it should be supervised and you should get feedback about them.'* (Interview 10, ST2)

**Figure 4.**
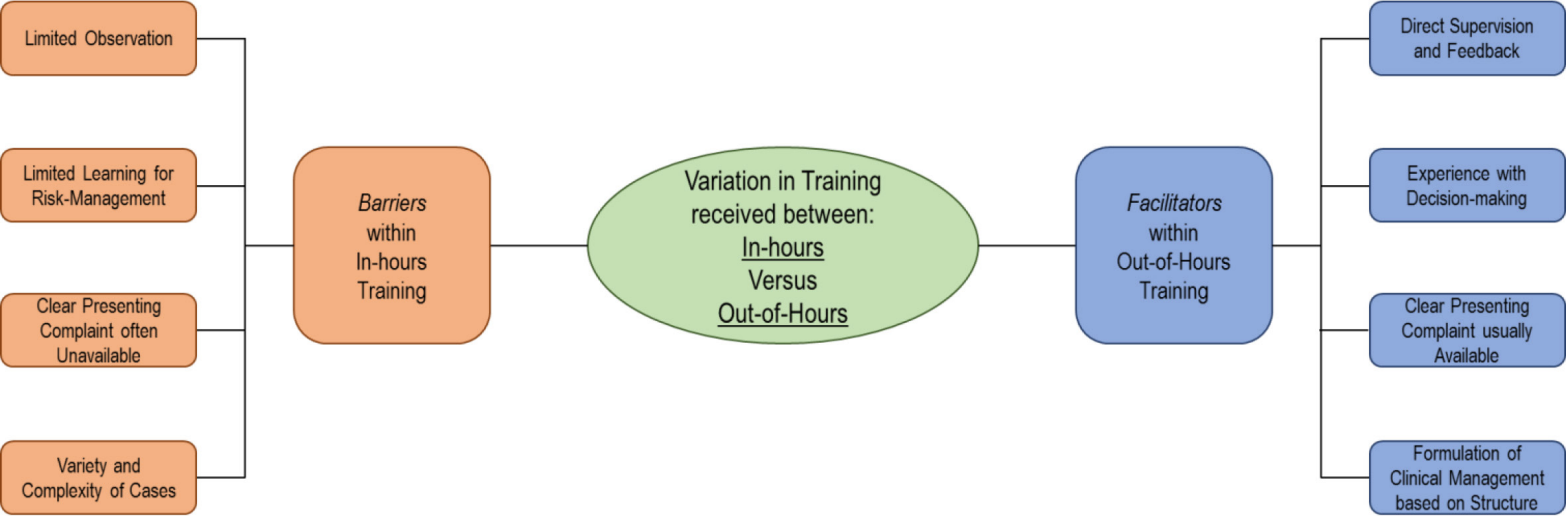
Factors shaping in-hours and out-of-hours training: barriers and facilitators.

This discrepancy can be explained by the different environments, particularly in relation to the potentially more complex and varied nature of in-hours telephone consultations (Figure 4).

**Table 2.** Relationship between in-hours and out-of-hours experience, with overall confidence, and satisfaction with supervision and feedback

	**Overall confidence in telephone consultations**		**Satisfaction with supervision received**		**Satisfaction with feedback received**	
	ρ	*P* value	ρ	*P* value	ρ	*P* value
***In-hours***Average number of months	0.65	<0.01	0.11	0.37	0.07	0.56
***In-hours***Average number of contacts per session	0.39	<0.01	<0.01	0.98	0.02	0.88
***Out-of-hours***Average number of months	0.52	<0.01	0.50	<0.01	0.56	<0.01
***Out-of-hours***Average number of contacts per session	0.58	<0.01	0.41	<0.01	0.49	<0.01

Spearman rank sum correlations were used to estimate ρ values.

## Discussion

### Summary

This mixed-methods study, involving a cross-sectional survey with follow-up semi-structured interviews, demonstrated that GP trainees felt least confident in independently undertaking more complex aspects of telephone consulting, and that there was a linear relationship between the training received and trainee confidence. Positive perceptions of telephone consultations were linked to managing trainee workload and perceived convenience for both patients and doctors, whereas negative experiences stemmed from more complex encounters, communication barriers, and inability to examine patients. Trainees felt that there were gaps in their competency acquisition and that the training for telephone consultations needs to be strengthened, but welcomed the recently introduced audio-COTs. The analyses also highlighted some differences in experiences between training in-hours and OOH, and identified key barriers and facilitators that explained the stronger association between training and satisfaction with supervision and feedback for OOH compared with in-hours training. Finally, the qualitative and quantitative findings can provide further understanding of telephone consultations for GP trainees, which may be useful for both trainees and trainers in both in-hours and OOH environments.

### Strengths and limitations

The strengths of this study include the focus on an under-investigated yet important (according to the participants) research topic. Involving both GP trainers and trainees in development of the questionnaire and interview schedule improved their validity. Using a mixed-methods approach that combined quantitative and qualitative methods allowed a richer understanding of the research area. The limitations of the study include potential lack of generalisability, since permission was only granted to contact trainees in one of three London regions. Therefore, experiences are dependent on local services and training priorities may be different elsewhere. There was a low response rate of 16%, typical of external healthcare surveys, which range from 15%–29%,^19^ despite several email reminders to trainees and announcements by programme directors. Third party provision of trainee contact details were relied on, which may not have been updated, artificially lowering the response rate. Demographic data for all NCEL GP trainees (both responders and non-responders) could not be provided, so it is difficult to establish whether the questionnaire population was reflective of the study population and examine generalisability directly. Furthermore, the first author was a GP trainee within one of the 11 VTS in the NCEL region. Although this helped with logistical aspects and allowed greater scrutiny of the project, it raises the issue of responder and researcher bias. There were, however, 100 responses and participation from all 11 VTS locations within NCEL with trainees at differing training stages, which helped to accommodate a wide range of experiences.

### Comparison with existing literature

To the authors' knowledge this is the first study investigating UK GP trainees’ experiences in telephone consultations. There are potential international synergies as well; for instance, in Australia, where remote consultations are common outside urban areas, a recent systematic review has highlighted a lack of high level evidence for the value of primary care telephone consultations when compared with face-to-face consultations.^20^ A number of recent studies have also highlighted a lack of training in telephone consultation-related competencies, with training suggested as a factor to minimise some of the potential adverse patient and medico-legal consequences associated with telephone consultations.^2,8^ Generic communication skills research shows that trainees recognise the importance of these skills in primary care and a preference for receiving feedback.^21^ The findings in relation to telephone consultations support this; demonstrating a strong preference towards supervisor observations, a current lack of feedback especially in routine hours, and the overall need to strengthen training.

The questionnaire highlighted the most common reason for positive experiences as being ‘efficiency in managing workload’, and this was further explained in the semi-structured interviews as supporting the capacity to take the *'* *history on the phone* *'*, and manage *'* *emergency problems* *'* remotely, thereby saving *'* *them the time of coming in* *'*. The literature highlights both positive and negative effects of telephone consultations on workload.^11,22,23^ The present study enhances these findings by adding the trainees’ points of view. Furthermore, although the study did not look specifically at patient satisfaction to telephone consultations,^24^ positive patient feedback was rated highly as one of the reasons for positive experiences. From a training perspective, audio-COTs also received positive feedback, and trainees felt that this implementation has been successful in providing structured feedback.^25^

There is limited evidence on telehealthcare-related education and training for practitioners.^26^ A recent telehealthcare review identified complex domains such as privacy and security as aspects that may become increasingly important, and that care for chronic conditions and medication reviews are important for telemedicine services.^27^ This study has demonstrated that complexity in consultations often left the trainees feeling under-confident, and that there was a positive correlation between the training received and the confidence to perform that particular competency independently. This has been demonstrated previously with respect to professional skills training in communicating with patients.^28,29^ While it is important to appreciate that confidence in specific attributes was correlated with the training received, this study does not assess patient-related outcomes, which are formed by clinical competence and performance.

Overall, the positive and negative experiences of trainees were broadly in line with those previously described for GPs in the literature. Patients have been shown to be highly satisfied in terms of ease of access, although opinions differ on the effect on workload, as highlighted within the analysis.^3,29^ It has been demonstrated that negative experiences stem from complex situations, absence of examination, difficulty in picking up cues and lack of specific training, and these findings are similar to known shortcomings of qualified GPs performing telephone consultations.^29^ From a policy perspective, telephone consultations are becoming more integral to practice,^5–7^ including OOH practice. GP trainees are also being introduced to different clinical situations, and changing clinical practice, within in-hours and OOH practice. Given the evolving landscape of primary health care and the potential diversity of the working environment, the comparison between these two fundamental environments is essential. The barriers and facilitators in OOH training when compared with in-hours training need further consideration, and this study provides important initial findings in this area.

### Implications for research and practice

With recent calls for *'* *more research assessing the effect of different training interventions on clinicians* *’* *telephone consultation skills* *'*,^8^ it has been shown that there appears to be a lack of specific training for GP trainees; and that overall feedback and supervision within this field needs strengthening. These findings, and the educational recommendations, are potentially relevant to the development of remote consulting curricula for postgraduate training worldwide, and specifically for the RCGP in the UK.^9,30^ They could also inform policymakers of the importance of training needs, as well as encouraging policymakers to consider both trainees' views in terms of the safe introduction of innovation in practice, and the views of practitioners on efficacy. The findings can also be reflected for the training development of other healthcare practitioners, not just GPs and trainees, who use telephone consultations and telemedicine.^5,31^ Future research might include replicating these analyses in other regions, and incorporating other modalities of telehealthcare, such as video and email consultations.

## References

[1] McLean S, Protti D, Sheikh A (2011). Telehealthcare for long term conditions. BMJ.

[2] van Galen LS, Car J (2018). Telephone consultations. BMJ.

[3] McKinstry B, Hammersley V, Burton C (2010). The quality, safety and content of telephone and face-to-face consultations: a comparative study. Qual Saf Health Care.

[4] Hobbs FDR, Bankhead C, Mukhtar T (2016). Clinical workload in UK primary care: a retrospective analysis of 100 million consultations in England, 2007–14. Lancet.

[5] NHS England (2015). Transforming primary care in London: a strategic commissioning framework.

[6] NHS England (2019). NHS Long Term Plan.

[7] NHS England (2019). Investment and evolution: A five-year framework for GP contract reform to implement The NHS Long Term Plan.

[8] Vaona A, Pappas Y, Grewal RS (2017). Training interventions for improving telephone consultation skills in clinicians. Cochrane Database Syst Rev.

[9] Royal College of General Practitioners (2016). The RCGP curriculum: professional & clinical modules. https://www.gmc-uk.org/-/media/documents/RCGP_Curriculum_modules_jan2016.pdf_68839814.pdf.

[10] Foster J, Jessopp L, Dale J (1999). Concerns and confidence of general practitioners in providing telephone consultations. Br J Gen Pract.

[11] Newbould J, Abel G, Ball S (2017). Evaluation of telephone first approach to demand management in English general practice: observational study. BMJ.

[12] Car J, Freeman GK, Partridge MR (2004). Improving quality and safety of telephone based delivery of care: teaching telephone consultation skills. Qual Saf Health Care.

[13] Sale JEM, Lohfeld LH, Brazil K (2002). Revisiting the quantitative-qualitative debate: implications for mixed-methods research. Qual Quant.

[14] Creswell JW, Plano Clark VL (2011). Designing and conducting mixed methods research.

[15] Pygall SA (2017). Telephone triage and consultation: are we really listening?.

[16] Braun V, Clarke V (2006). Using thematic analysis in psychology. Qual Res Psychol.

[17] Braun V, Clarke V, Hayfield N, Terry G, Liamputtong P (2019). Thematic analysis. Handbook of research methods in social sciences.

[18] Clarke V, Braun V (2013). Teaching thematic analysis: overcoming challenges and developing strategies for effective learning. Psychologist.

[19] Coulter A, Locock L, Ziebland S (2014). Collecting data on patient experience is not enough: they must be used to improve care. BMJ.

[20] Downes MJ, Mervin MC, Byrnes JM (2017). Telephone consultations for general practice: a systematic review. Syst Rev.

[21] Van Nuland M, Thijs G, Van Royen P (2010). Vocational trainees' views and experiences regarding the learning and teaching of communication skills in general practice. Patient Educ Couns.

[22] Bunn F, Byrne G, Kendall S (2004). Telephone consultation and triage: effects on health care use and patient satisfaction. Cochrane Database Syst Rev.

[23] Holt TA, Fletcher E, Warren F (2016). Telephone triage systems in UK general practice: analysis of consultation duration during the index day in a pragmatic randomised controlled trial. Br J Gen Pract.

[24] Caralis P (2010). Teaching residents to communicate: the use of a telephone triage system in an academic ambulatory clinic. Patient Educ Couns.

[25] Sales B, Scallan S, Crane S (2015). The audio-COT (consultation observation tool): developing a new assessment tool for GP training. Educ Prim Care.

[26] Edirippulige S, Armfield NR (2017). Education and training to support the use of clinical telehealth: a review of the literature. J Telemed Telecare.

[27] Tuckson RV, Edmunds M, Hodgkins ML (2017). Telehealth. N Engl J Med.

[28] Noble LM, Kubacki A, Martin J (2007). The effect of professional skills training on patient-centredness and confidence in communicating with patients. Med Educ.

[29] Car J, Sheikh A (2003). Telephone consultations. BMJ.

[30] RCGP (2019). GP curriculum: overview. https://www.rcgp.org.uk/training-exams/training/gp-curriculum-overview.aspx.

[31] NHS England (2016). General practice forward review. https://www.england.nhs.uk/wp-content/uploads/2016/04/gpfv.pdf.

